# Time-Domain and Spectral-Domain Optical Coherence Tomography of Retinal Nerve Fiber Layer in MS Patients and Healthy Controls

**DOI:** 10.1155/2012/564627

**Published:** 2012-05-22

**Authors:** Alex P. Lange, Reza Sadjadi, Jameelah Saeedi, Janette Lindley, Fiona Costello, Anthony L. Traboulsee

**Affiliations:** ^1^Neuro-Ophthalmology Division, Department of Ophthalmology, Eye Care Center (VGH), The University of British Columbia, 2550 Willow Street, Vancouver, BC, Canada V5Z 3N9; ^2^Division of Neurology, Department of Medicine, University of British Columbia, S199 Koerner Pavillion, Wesbrook Mall, Vancouver, BC, Canada V6T 2B5; ^3^Departments of Clinical Neurosciences and Surgery and Hotchkiss Brain Institute, University of Calgary, Calgary, AB, Canada T2N 1N4

## Abstract

*Objective*. The aim of this study was to compare retinal nerve fiber layer thickness (RNFLT) between spectral-domain (SD-) and time-domain optical coherence tomography (TD-OCT) in MS patients and healthy controls (HC). Furthermore, RNFLT between MS eyes with and without optic neuritis (ON) and HC should be explored. Finally, the relationship between RNFLT, disease duration, EDSS, and disease modifying therapy (DMT) should be established. *Design*. Prospective, cross-sectional study. *Participants*. 28 MS patients and 35 HC. *Methods*. Both groups underwent TD- and SD-OCT measurements. RFNLT was correlated between the two machines and between MS eyes with and without ON and HC. Furthermore, RNFLT was correlated to disease duration, EDSS and DMT. *Results*. A strong correlation (Pearson's *r* = 0.921, *P* < 0.001), but a statistically significant difference of 2 *μ*m (*P* < 0.001), was found between the two devices. RNFLT was significantly different between MS eyes with history of ON (mean RFNLT (SD) 72.21 *μ*m (15.83 *μ*m)), MS eyes without history of ON 93.03 *μ*m (14.25 *μ*m), and HC 99.07 *μ*m (7.23 *μ*m) (*P* < 0.001). *Conclusions*. The measurements between different generation of OCT machines are not interchangeable, which should be taken into account if comparing results between different machines and switching OCT machine in longitudinal studies.

## 1. Introduction

Optical coherence tomography (OCT) is a noninvasive technique for high-resolution, cross-sectional tomographic imaging of retinal tissue using backscattered light. OCT imaging is very similar to ultrasound B-Scan imaging but uses infrared-light instead of ultrasound waves. Two-dimensional, cross-sectional images are obtained from multiple axial scans (A-Scans) at different transverse locations [1].

Until recently, third-generation time-domain OCT (TD-OCT) using Stratus OCT (Carl Zeiss Meditec AG, Jena, Germany) has been widely used to acquire images at a rate of 400 axial scans per second with an axial resolution of 10 *μ*m [2]. The recently introduced fourth-generation spectral-domain OCT (SD-OCT) has improved depth resolution by a factor of three (axial resolution up to 3.8 *μ*m) and allows a significantly higher acquisition speed (40'000 axial scans per second) resulting in improved image quality and minimized motion artefacts [3]. Furthermore, software improvements allow reconstruction of a three-dimensional image of the retina.

Heidelberg Spectralis OCT (Heidelberg Engineering, Heidelberg, Germany) uses an integrated eye tracking system (IETS) to compensate for eye movement artefact during data acquisition. IETS also allows an automatic re-centration, which can be used for more reliable follow-up scans. Heidelberg noise reduction technology helps producing significantly improved images by adjusting data and reducing noise using mean values from several scans [4]. Heidelberg Spectralis OCT needs to be validated for accuracy, reproducibility, and comparability to previous models before it can be reliably used for clinical and research purposes. 

Recent studies have shown differences between SD-OCT machines (Cirrus SD-OCT, Carl Zeiss Meditec AG, Jena, Germany [5–10], RTVue-100, Optovue Inc., Fremont, CA, USA [11], and Spectralis, Heidelberg Engineering, Heidelberg, Germany, [12, 13]) and TD-OCT in healthy controls and glaucoma patients. These studies showed better reproducibility compared to TD-OCT and significant differences in RNFLT measurements between the two generations of machines.

So far only few studies have examined the role of SD-OCT in multiple sclerosis (MS) patients [14, 15]. Therefore, our specific study aims are (1) to compare retinal nerve fiber layer thickness (RNFLT) measurements between the validated third-generation Stratus OCT and the new fourth-generation Heidelberg Spectralis OCT in MS patients and healthy controls, (2) to compare RNFLT between MS eyes affected by optic neuritis (ON eyes) to eyes without a history of ON (NON eyes) and control eyes, and finally (3) to determine the relationship between RNFLT, disease duration, expanded disability status scale (EDSS), and disease-modifying therapy (DMT) in MS patients and refraction in both groups.

## 2. Materials and Methods

### 2.1. Study Design and Patient Population

In a prospective, cross-sectional study, subjects with MS and controls were identified from the UBC Hospital MS Clinic with the aid of advertisement and pamphlets. All MS subjects had confirmed diagnosis of MS made by a neurologist with specific experience in managing MS patients, and based on the modified McDonald criteria [16].

### 2.2. Inclusion and Exclusion Criteria

Patients with a recent history of optic neuritis (ON) (<6 months), history of ocular diseases (age-related macular degeneration, diabetic retinopathy, uveitis, and glaucoma), and history of other diseases that could mimic MS or affect OCT testing (neuromyelitis optica, parkinson's disease, and Alzheimer disease) and subjects with difficulties maintaining fixation were not included.

### 2.3. Outcome Measures

#### 2.3.1. Clinical Data

Clinical history information such as disease duration from time of disease onset, previous history of optic neuritis, and other neurological information like EDSS score was obtained by history and from hospital charts after patient recruitment. Myopia was defined as spherical equivalent of <−0.50 diopters, emmetropia between −0.5 and +0.5 diopters, and hyperopia as >+0.5 diopters measured by SD-OCT. 

#### 2.3.2. Optical Coherence Tomography

OCT was performed in a random order by an experienced person that was masked to clinical data, using TD-OCT and SD-OCT within one-hour period with no pupil dilation (half of subjects had TD-OCT prior to SD-OCT and vice versa).

TD-OCT (Stratus OCT 3000, Software Version 4.0.7; Carl Zeiss Meditec, Jena, Germany): the standard Fast RNFL acquisition protocol was used. Three scans, each composed of 256 A scans, were automatically acquired consecutively using a circle scan with a standardized diameter of 3.4 mm by the same experienced operator. Several scans were taken and the best-centered scan with a quality score of ≥6 was chosen for analysis (as suggested by the manufacturer). An automated computer algorithm delineated the anterior and posterior margins of the RNFL.

SD-OCT (Heidelberg Spectralis OCT, Software Version 5.1.2, Heidelberg Engineering, Heidelberg, Germany): The RNFL protocol in high-resolution mode (axial resolution 3.8 *μ*m, 19'000 scans per second) was used. Sixteen consecutive circular B-scans (each composed of 1536 A scans) with a diameter of 3.4 mm were automatically averaged to reduce speckle noise. The online tracking system compensated for eye movements. Several scans were taken by the same experienced operator and the best centered with a quality of at least 24 (which is about the equivalent of 6 in Stratus OCT) was chosen for analysis. The included software algorithm delineated the anterior and posterior margins of the RNFL.

### 2.4. Statistical Analysis

Microsoft Office 2007 and SPSS Version 16.0 for Windows were used to do statistical analysis. Descriptive, mean comparison (*t*-test and one-way ANOVA) and correlation analysis (Pearson's) were used to compare OCT measures between different groups: SD-OCT versus TD-OCT RNFLT measurements; MS eyes versus control eyes; myopic versus emmetropic and hyperopic eyes. *P* values less than 0.05 were considered to be statistically significant.

## 3. Results

### 3.1. Patient Demographics and Clinical Characteristics

The study recruitment took place between August 2009 and February 2010. Twenty-eight MS patients (age mean: 38.88 yrs; SD: 11.65 yrs, mean disease duration: 83.12 months, SD: 83.76; 25 with relapsing-remitting and 3 with secondary progressive MS; EDSS range between 1.5 and 6.5, mean: 2.8, SD: 1.6) and 35 healthy controls (age mean: 43.46 yrs; SD: 9.08 yrs) participated in this study (Table 1). Fourteen (out of 27) patients used DMT for MS. Sixteen (out of 27) patients had an EDSS score of less than 3.0. All subjects were examined by SD-OCT and TD-OCT machines. Two subjects were excluded in the control group due to (1) software failure to delineate RNFL correctly and (2) OCT artefacts due to high myopia. One patient in the MS group was excluded due to inability to measure exact refraction after refractive surgery. Out of the remaining 120 eyes 32 eyes were myopic (refraction range between −8.25 and −0.75 diopters), 74 eyes were emmetropic (refraction = 0 diopters), and 14 eyes were hyperopic (refraction range between +1 and +6 diopters). Fourteen (out of 54) MS eyes were previously affected by a single optic neuritis event.

### 3.2. Comparing Time-Domain and Spectral-Domain OCT

**Figure 1:**
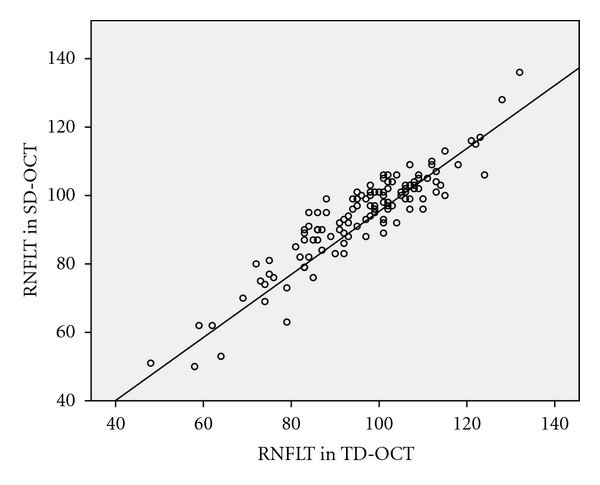
Correlation between average RNFLT in *μ*m in SD-OCT (*y*-axis) and TD-OCT (*x*-axis).

SD-OCT and TD-OCT RNFLT values were strongly correlated in all quadrants with correlation coefficient ranging from 0.808 (*P* < 0.01) in inferior quadrant to 0.878 (*P* < 0.01) in temporal quadrant. The overall RNFLT was also strongly correlated (correlation coefficient = 0.921; *P* < 0.001) between the two machines (Figure 1). However, RNFLT values showed minor but statistically significant differences between the two machines (*P* < 0.001) (Table 2).

### 3.3. Comparing RNFLT Measurements between MS and Control Eyes

**Figure 2:**
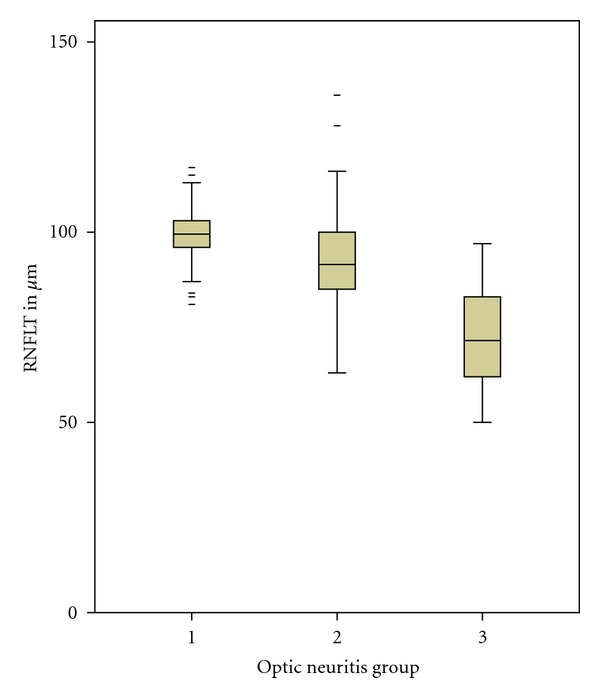
Boxplot of differences between RNFLT in control group (left box, group 1) and groups of MS eyes without optic neuritis (center box, group 2) and MS eyes with optic neuritis (left box, group 2) measured by SD-OCT.

Overall, MS patients had significantly lower RNFLT measured by both SD-OCT and TD-OCT (Table 3). Moreover, RNFLT was significantly different between MS eyes with history of optic neuritis (mean RFNL (SD) 72.21 *μ*m (15.83 *μ*m)), MS eyes without history of optic neuritis 93.03 *μ*m (14.25 *μ*m), and healthy controls 99.07 *μ*m (7.23 *μ*m) (*P* < 0.001) measured by SD-OCT (Figure 2).

### 3.4. Correlation between Retinal Nerve Fiber Layer Thickness, Disease Duration, EDSS, DMT, and Refraction

When all MS eyes were considered, duration of the disease since onset of first symptoms weakly correlated with superior (*r* = −0.28; *P* = 0.048) and temporal (*r* = −0.33; *P* = 0.02) quadrant RNFLT. There was no significant correlation between disease duration and mean RNFLT values (*r* = −0.13; *P* = 0.35), or RNFLT values in the inferior (*r* = −0.14; *P* = 0.31) or nasal (*r* = −0.19; *P* = 0.17) quadrants. When only MS eyes without a history of ON were considered, a moderate correlation (*r* = −0.44; *P* = 0.01) was found between mean RNFLT and disease duration, with a significant correlation in the superior and inferior quadrants (*r* = −0.51; *P* = 0.001, and *r* = −0.38; *P* = 0.02, resp.) and no significant correlation in the temporal and nasal quadrants (*P* = 0.91 and *P* = 0.08, resp.).

When EDSS was correlated to mean RNFLT in all MS eyes, a weak correlation was found (*r* = − 0.3; *P* = 0.05). This was significant in the superior quadrant only (*r* = −0.33; *P* = 0.02), nonsignificant in the other quadrants (*P* = 0.46 for temporal quadrant, *P* = 0.07 for inferior quadrant, and *P* = 0.39 for nasal quadrant). When only MS eyes without a history of ON were considered, a moderate correlation was found between EDSS and mean RNFLT (*r* = −0.35; *P* = 0.03). This was also significant in the superior (*r* = −0.38; *P* = 0.02) and inferior quadrants (*r* = −0.33; *P* = 0.05), but not in the temporal (*P* = 0.31) and nasal quadrants (*P* = 0.43). 

The minimal difference between RFNLT in patients with DMT (86.07 *μ*m) and without DMT (90.82 *μ*m) was not statistically different (*P* = 0.327).

Myopic and emmetropic eyes showed significantly different RFNLT measurements in both SD-OCT (mean RNFLT (SD) 87.75 *μ*m (12.52 *μ*m) versus 96.46 *μ*m (14.61 *μ*m)) and TD-OCT (89.59 *μ*m (13.65 *μ*m) versus 99.19 *μ*m (15.11 *μ*m)). There was a significant correlation between refraction in diopters and RNFLT (*r* = 0.4; *P* = 0.005). 

## 4. Discussion

The main aim of this study was to compare RNFLT measurements between SD-OCT and TD-OCT in MS patients and healthy controls. Our results show strong correlations (Pearson's *r* = 0.921) between the measurements of the Heidelberg Spectralis SD-OCT and TD-OCT. RNFLT values were significantly lower in SD-OCT (mean difference 2 *μ*m). These results are similar to those of Watson et al. [14], who found Spectralis SD-OCT to measure 3 *μ*m lower than TD-OCT in a study of 50 MS eyes, and Seibold et al. [12], who found the same results in a series of 80 healthy eyes. On the other hand, Arthur et al. [13] compared Spectralis SD-OCT with TD-OCT in 30 healthy eyes and found Spectralis to measure 6 *μ*m lower, a larger difference than our results. Studies using other SD-OCT devices showed similar discrepancies with Cirrus SD-OCT measuring lower RNFLT and RTVue measuring higher RNFLT than TD-OCT (Table 4).

The discrepancy between different devices may be explained by a difference in calibration due to a higher resolution and improved software algorithm in more recent models [6]. This has been addressed in evaluation of SD-OCT in macular thickness [17, 18]. The phenomenon of thickness-dependent interdevice differences was not observed in our data [15]. The minimal difference observed in our study is lower than the axial resolution of the SD-OCT, hence clinically not significant. However, results from these machines cannot be interchangeably interpreted in a population study and ongoing longitudinal studies switching generation of OCT should take these differences into consideration. 

There has been increasing interest in RNFLT measurements in MS patients in order to determine whether OCT can be used as a surrogate marker for follow-up examinations. Therefore, a large amount of cross-sectional data has been previously published. Many studies have shown differences between RNFLT in MS eyes with optic neuritis, MS eyes without optic neuritis, and healthy controls, for example, [15, 19–27]. All these studies were using the older TD-OCT technology. We were able to reproduce these differences using the newer generation of OCT machine. Spectral-domain OCT has several advantages over the older TD-OCT technology: there is no pupil dilation needed, the speed of the machines is higher, reducing the possibilities of motion artefacts, and the lack of the previously used bright flashlight makes the examination much more comfortable for the patient. Furthermore, the higher resolution and the improved software algorithm allowing automatic re-centration for follow-up exams help in improving accuracy and reproducibility for follow-up exam in longitudinal studies. Up to date, no longitudinal study in an MS cohort has been published using SD-OCT technology. Two longitudinal studies using TD-OCT have not been able to show any change in RNFLT in a two-year follow-up period [27, 28]. Only Talman et al. [21] could detect significant RNFLT changes in a 4.5-year study of 299 patients using TD-OCT (loss of 2.9 *μ*m at 2 to 3 years and 6.1 *μ*m at 3 to 4.5 years; *P* < 0.001). This pattern was observed in both eyes with and without history of ON. Proportions of eyes with RNFL loss greater than test-retest variability (≥6.6 *μ*m) increased from 11% at baseline to 44% at final visit (3–4.5 years) (*P* < 0.001). The progressive axonal loss of approximately 2 *μ*m per year could only be detected over a relatively long period of time. This is most likely due to the relatively low resolution of the TD-OCT machine. The new generation SD-OCT is more sensitive to smaller changes and may be more reliable detecting RNFL changes over shorter time periods. Longitudinal studies using SD-OCT technology will be needed to establish if OCT measurements can be used as a surrogate marker in MS and be used to monitor disease progression and disease-modifying therapy. 

We were interested in contribution of disease duration, EDSS, refraction, and status of DMT on RNFLT measurements. Our results showed no significant correlation for disease duration and EDSS when all MS eyes were compared but moderate correlation when only eyes without a history of ON were considered. This may be due to the fact that ON causes a 18–22% loss of RNFLT and the small progressive loss of RNFLT is not evident at this time anymore [29]. The difference between patients with and without DMT was statistically not significant, but our sample size was too small for a final conclusion. 

We also compared RNFLT measurements between refraction range groups (described in methods). We showed a relatively large difference between myopic and nonmyopic eyes using both devices. Thinner RNFLT measurements in myopes may be explained by increased scan diameter due to the telecentric optics of the OCT in increased myopia and myopic tilted discs resulting in elevated and decreased RNFLT at different sites. Furthermore, the centration is very difficult even on a frozen fundus image due to the asymmetry of the disc. 

RNFLT values were significantly lower in myopic eyes as the diameter of the scan increases with higher myopic refraction. Rauscher et al. [30] have reported an average decrease of RNFL of 3 *μ*m per diopter of myopia. A possible explanation is the telecentric system, which keeps the angle of the OCT beam constant at 12 degrees. In our measurements, the scan diameter increased to 3.8 mm in −5 diopters and to 4.2 mm in −10 diopters. This results in thinning of about 10 *μ*m in −5 diopters and about 20 *μ*m in −10 diopters [31]. This was not a major issue with the older TD-OCT as the axial resolution is only 10 *μ*m but gets more importance with the SD-OCT devices with higher resolution up to 3.8 *μ*m. This must be taken into consideration designing future studies. Higher myopic refraction should be excluded or properly matched between groups. Furthermore, normative databases are needed to be refraction adjusted. 

The main aim of the study was not to characterize RNFLT in MS population. Therefore, the MS population involved was randomly selected and examiner was not blinded to subjects' diagnosis and history of optic neuritis. Furthermore, the groups were not gender- or age-matched and both eyes of each subject were included. However, this was not a major issue in comparing RNFLT in the same subject between two different machines and did not affect the results of our main study aim.

## Figures and Tables

**Table 1:** Descriptive statistics of MS and control group.

	Control group	MS group
*N*	35	28
Mean age in years (±SD)	38.88 (11.65)	43.46 (9.08)
Gender	15 female 20 male	23 female 5 male
Number excluded	2	1
Average RNFL SD-OCT (micrometer ± SD)	98.59 (6.74)	88.80 (17.39)
Average RNFL TD-OCT (micrometer ± SD)	100.67 (8.88)	90.91 (18.09)
Number of eyes with optic neuritis	n/a	14 (26%)
Number of patients with SPMS	n/a	3 (11%)
Mean EDSS (SD)	n/a	2.8 (1.6)
Mean disease duration in months (SD)	n/a	83.12 (83.67)
On disease-modifying therapy	n/a	52% (14/27)

**Table 2:** Differences in RNFLT between SD- and TD-OCT in MS group and control group (SD-OCT minus TD-OCT).

	Control group		MS group	
	Mean difference in *μ*m (95% CI)	Pearson's Corr *r*	Mean difference in *μ*m (95% CI)	Pearson's Corr *r*
Average	−2.40 (−3.70 to −1.09)	0.83 (*P* < 0.001)	−1.69 (−3.44 to 0.06)	0.93 (*P* < 0.001)
Superior	3.75 (0.99 to 6.51)	0.77 (*P* < 0.001)	5.54 (2.20 to 8.87)	0.90 (*P* < 0.001)
Temporal	0.04 (−1.41 to 1.50)	0.88 (*P* < 0.001)	−0.54 (−3.02 to 1.94)	0.87 (*P* < 0.001)
Inferior	−6.56 (−8.28 to −4.84)	0.85 (*P* < 0.001)	−6.81 (−9.51 to −4.11)	0.91 (*P* < 0.001)
Nasal	−6.93 (−9.47 to −4.38)	0.80 (*P* < 0.001)	−3.37 (−7.13 to 0.40)	0.81 (*P* < 0.001)

**Table 3:** Overview RNFLT between SD- and TD-OCT in MS and control groups, separated by quadrants.

	SD-OCT *μ*m mean (SD)		TD-OCT *μ*m mean (SD)	
	Control	MS	Control	MS
Average	98.59 (6.79)	88.80 (17.55)	100.67 (8.96)	90.9107 (18.26)
Superior	121.27 (16.22)	107.25 (26.95)	117.48 (16.87)	101.88 (27.04)
Temporal	71.17 (10.88)	65.02 (15.92)	70.86 (12.88)*	65.82 (17.57)*
Inferior	127.16 (13.10)	114.80 (21.64)	133.23 (13.59)	120.36 (27.08)
Nasal	74.55 (11.21)	68.07 (21.53)	81.031 (15.75)	72.20 (24.34)

*All measures are significantly different between MS patients and control except for temporal quadrant measured by TD-OCT.

**Table 4:** Overview published studies comparing RNFLT in SD-OCT versus TD-OCT.

Author	SD-OCT used	Study population (eyes)	Results
Chang et al. [5]	Cirrus	54 glaucoma 50 controls	Cirrus is equivalent to Stratus for detecting glaucoma
Knight et al. [6]	Cirrus	101 glaucoma 29 controls	Cirrus 7 *μ*m lower then Stratus in both groups
Leung et al. [7]	Cirrus	83 glaucoma 97 controls	Cirrus 12 *μ*m lower for control, 6 *μ*m lower in glaucoma
Sung et al. [8]	Cirrus	103 glaucoma 60 controls	Cirrus 13 *μ*m lower for control, 14 *μ*m lower in glaucoma
Vizzeri et al. [9]	Cirrus	78 glaucoma 32 controls	Cirrus 8 *μ*m lower for control, 6 *μ*m lower in glaucoma
Kim et al. [10]	Cirrus	27 controls	Cirrus 10 *μ*m lower
Gonzalez-Garcia et al. [11]	RTVue-100	76 glaucoma 60 controls	RTVue 2 *μ*m higher
Seibold et al. [12]	Cirrus, Spectralis, RTVue-100	80 controls	Spectralis 3 *μ*m lower, Cirrus 12 *μ*m lower, RTVue 3 *μ*m higher,
Arthur et al. [13]	Spectralis	30 controls	Spectralis 6 *μ*m lower
Watson et al. [14]	3D OCT-1000, Cirrus, RTVue-100, Spectralis	50 MS	Spectralis 3 *μ*m lower, Cirrus 8 *μ*m lower, RTVue 3 *μ*m higher, 3D OCT-1000 2 *μ*m higher
Bock et al. [15]	Cirrus	110 MS	Cirrus 8 *μ*m lower
